# Biomarker analysis of the NeoSphere study: pertuzumab, trastuzumab, and docetaxel versus trastuzumab plus docetaxel, pertuzumab plus trastuzumab, or pertuzumab plus docetaxel for the neoadjuvant treatment of HER2-positive breast cancer

**DOI:** 10.1186/s13058-017-0806-9

**Published:** 2017-02-09

**Authors:** Giampaolo Bianchini, Astrid Kiermaier, Giulia Valeria Bianchi, Young-Hyuck Im, Tadeusz Pienkowski, Mei-Ching Liu, Ling-Ming Tseng, Mitch Dowsett, Lila Zabaglo, Sarah Kirk, Tania Szado, Jennifer Eng-Wong, Lukas C. Amler, Pinuccia Valagussa, Luca Gianni

**Affiliations:** 1 Oncologia Medica, Ospedale San Raffaele, Milan, Italy; 2 Genentech, Inc., Basel, Switzerland; 3 Oncologia Medica 1, Fondazione IRCCS Istituto Nazionale dei Tumori, Milan, Italy; 4 Samsung Medical Center, Seoul, Korea; 5 Postgraduate Medical Education Center, Warsaw, Poland; 6 Koo Foundation Sun Yat-Sen Cancer Center, Taipei, Taiwan, Republic of China; 7 Taipei-Veterans General Hospital, National Yang-Ming University, Taipei, Taiwan, Republic of China; 8 Centre for Molecular Pathology, Royal Marsden Hospital, London, UK; 9 Institute of Cancer Research, London, UK; 10 Roche Products Limited, Welwyn, UK; 11 Genentech, Inc., South San Francisco, CA USA; 12 Fondazione Michelangelo, Milan, Italy

**Keywords:** Biomarker, Breast cancer, Docetaxel, HER2, Neoadjuvant, Pertuzumab, Trastuzumab

## Abstract

**Background:**

NeoSphere showed significantly higher pathologic complete response (pCR) with neoadjuvant pertuzumab, trastuzumab, and docetaxel compared with trastuzumab plus docetaxel, pertuzumab plus trastuzumab, or pertuzumab plus docetaxel. We assessed associations between human epidermal growth factor receptor 2 (HER2) pathway-related biomarkers and clinical outcome in response to these regimens.

**Methods:**

Tumor, serum, and whole blood samples were collected at baseline and post neoadjuvant treatment before surgery. Associations between biomarkers and pCR, and between biomarkers and clinical variables were assessed in the overall and estrogen receptor (ER)-positive and ER-negative populations. Changes in serum marker levels between baseline and post-neoadjuvant treatment were examined.

**Results:**

No markers were associated with pCR across all groups; however, significant associations were observed for two markers in individual groups. High HER2 was significantly associated with higher pCR rates (*P* = 0.001) and a significant treatment interaction (*P* = 0.0236) with pertuzumab, trastuzumab, and docetaxel (odds ratio 2.07, *P* = 0.01). Low serum transforming growth factor alpha (TGFα) was associated with higher pCR rates with pertuzumab plus trastuzumab (*P* = 0.04) without a significant treatment interaction. Presence of truncated HER2 did not affect pCR. A non-significant decreased pCR benefit was observed consistently across groups in patients with mutated *PIK3CA* while the treatment benefit from pertuzumab was maintained when comparing the trastuzumab plus docetaxel and pertuzumab, trastuzumab, and docetaxel groups. Notably, *PIK3CA* exon 9 mutations were associated with residual disease (pooled groups), which was not found for exon 20 mutations. Serum HER2 extracellular domain levels were significantly increased between baseline and post-neoadjuvant treatment in the non-trastuzumab-treated group, and decreased in the trastuzumab-containing groups (likely due to trastuzumab’s mechanism of action). Differences in biomarker profiles according to ER status were observed.

**Conclusions:**

The observed associations of HER2 protein levels with sensitivity to pertuzumab, and of *PIK3CA* exon 9 mutation to lack of sensitivity to HER2-targeted monoclonal antibody treatment, warrant further investigation. Previously reported findings of truncated forms of HER2 as resistance markers to HER2-targeted treatment could not be confirmed in NeoSphere. Conventional HER2 assessment should continue and HER2 remains the only biomarker suitable for patient selection in this population.

**Trial registration:**

Clinicaltrials.gov, NCT00545688. Registered on 16 October 2007.

**Electronic supplementary material:**

The online version of this article (doi:10.1186/s13058-017-0806-9) contains supplementary material, which is available to authorized users.

## Background

Human epidermal growth factor receptor 2 (HER2) is the clinically validated biomarker for HER2-targeted therapies, several of which are approved in the neoadjuvant, adjuvant, and metastatic settings for HER2-positive breast cancer. Pertuzumab (PERJETA^®^, F. Hoffmann-La Roche Ltd., Basel, Switzerland) is directed at the dimerization domain of HER2 and inhibits dimerization with other HER receptors while stimulating antibody-dependent cell-mediated cytotoxicity (ADCC) [1–3]. Trastuzumab (Herceptin^®^, F. Hoffmann-La Roche Ltd.) binds to the transmembrane domain to inhibit mitogenic signaling, block HER2 cleavage, and stimulate ADCC [4–6]. Due to their different binding modalities, trastuzumab and pertuzumab have complementary proposed mechanisms of action [7].

In the NeoSphere study, patients treated with neoadjuvant pertuzumab, trastuzumab, and docetaxel (Taxotere^®^, Sanofi-Aventis, Paris, France) (group B) had a significantly higher pathologic complete response (pCR) rate compared with those treated with trastuzumab plus docetaxel (group A), pertuzumab plus trastuzumab (group C), or pertuzumab plus docetaxel (group D) (45.8% versus 29.0%, 16.8%, and 24.0%, respectively) [8]. The combination of pertuzumab, trastuzumab, and docetaxel has also been shown to be superior to trastuzumab plus docetaxel in the first-line treatment of metastatic breast cancer [9, 10]. The combination is now approved by the European Medicines Agency (EMA) and the US Food and Drug Administration (FDA) in both settings.

HER2-positive/estrogen receptor (ER)-positive and HER2-positive/ER-negative breast cancer are known to have different patterns of gene expression, and treatment outcome seems to be driven by different biologic pathways [8, 11–14]. Hormone-receptor-negative status was associated with an increase in treatment benefit with pertuzumab plus trastuzumab when groups A and B were compared in NeoSphere [8]. It is known that HER2 expression in ER-negative, HER2-positive tumors is higher than that in ER-positive, HER2-positive tumors [15].

We assessed a panel of biomarkers, in tumor specimens and in sera, to characterize the molecular profile of the biomarker population of NeoSphere, and to explore biomarkers correlated with different treatments and/or different subsets of patients in this trial. The objective of this exploratory analysis was to identify those biomarkers (or combinations of biomarkers) with the best association (positive or negative) with pCR in response to the treatment regimens used in the NeoSphere study. In particular, we attempted to identify biomarkers that would predict a benefit from the addition of pertuzumab to a trastuzumab-based regimen. The markers tested included those that are involved in downstream signaling of HER2, belong to a group of related receptor tyrosine kinases that could serve as salvage routes for an inhibited HER2 pathway, or are ligands of HER family proteins that induce activation of the HER2 pathway. We also assessed the presence of truncated forms of HER2, including p95^HER2^, with regards to pCR, as p95 was previously reported to predict worse outcomes in patients with HER2-positive breast cancer [16].

## Methods

### Study design and patients

The study design and patient characteristics have been reported previously [8]. Briefly, NeoSphere (NCT00545688) [17] was a multicenter, open-label, phase II study. Treatment-naive women with HER2-positive breast cancer were randomized (1:1:1:1) centrally and stratified by operable, locally advanced, and inflammatory breast cancer, and by hormone receptor expression, to receive four neoadjuvant cycles of study treatment. The clinical endpoint was pCR, defined as the absence of invasive neoplastic cells at microscopic examination of the primary tumor at surgery. Remaining *in situ* lesions were allowed [8].

### Specimen characteristics

Collection of core biopsies, sera, and whole blood from all patients was mandatory at baseline. Tumor samples were obtained as formalin-fixed, paraffin-embedded tissue. Tissue obtained after the neoadjuvant treatment period was derived from resection specimens.

### Assay methods

Tissue processing, immunohistochemistry (IHC), fluorescence *in situ* hybridization (FISH), RNA extraction, quantitative reverse transcription polymerase chain reaction (qRT-PCR), and DNA isolation were performed centrally by Targos Molecular Pathology GmbH, Kassel, Germany. Targos followed the protocols and processing instructions developed by Roche Diagnostics GmbH, Penzberg, Germany. Commercially available assays or kits were used where specified, and performed according to the manufacturer’s instructions. All other assays were developed by Roche Diagnostics for exploratory research purposes only.

Expression of HER2 (HercepTest, Dako, Glostrup, Denmark), HER3 (HER3 M7297, Dako), insulin-like growth factor 1 receptor (IGF1R, Clon 1.004.168, Roche Diagnostics), phosphatase and tensin homolog (PTEN, AF847, R&D Systems, Minneapolis, MN, USA), and pAKT (#3787, Cell Signaling Technology, Danvers, MA, USA) were assessed by IHC, and a modified H-score [18] was derived for each marker. The modified H-score was calculated as the percentage of cells stained per intensity level, multiplied by a factor composed of the intensity category plus 1:

$$ \mathrm{Modified}\ \mathrm{H}\hbox{-} \mathrm{score} = \left(1 + 1\right) \times \mathrm{P}1 + \left(2 + 1\right) \times \mathrm{P}2 + \left(3 + 1\right) \times \mathrm{P}3 $$

Therefore the modified H-score has a maximum value of 400 instead of the standard H-score of 300. The percentage of cells stained with an intensity of 0 was used only for quality control. Cases with no staining on the tissue section were assigned a score of 0. Modified H-scores were calculated for subcellular compartments for which specific staining was identified and for which there was a biologic rationale for the subcellular location of the respective marker (e.g. for PTEN and AKT nuclear staining).

Image acquisition and analysis of HER2 staining intensity took place at the Royal Marsden Hospital (London, UK) using the Ariol image analysis system (Leica Microsystems (Gateshead) Ltd., UK) equipped with a BX61 microscope (Olympus, Southend-on-Sea, UK) and a black and white MegaPlus ES 4.0/E camera (Redlake MASD, Inc., San Diego, CA, USA). Slides were scanned and analyzed as previously described [19], except that five representative invasive breast cancer areas in each image were selected. The mean membrane intensity of all five representative areas selected for analysis was used as a measurement of HER2 staining intensity.

*HER1*, *HER2*, *HER3*, *Amphiregulin* (*AREG*) and *Betacellulin* mRNA levels in tumor tissue were assessed relative to the *G6PD* gene by qRT-PCR (Roche Diagnostics, research-only assay). *c-Myc* amplification was assessed by FISH (MYC/CEN-8 FISH Probe Mix, Dako).

Mutational analyses of eight mutations at four hot spots in exons 7, 9, and 20 within the gene encoding the catalytic subunit of phosphoinositide 3-kinase (*PIK3CA*) on tumor DNA were performed using TaqMan-PCR (Roche Diagnostics, research-only assay) at Roche Translational Research Sciences (TRS, Basel, Switzerland). This led to a technical success rate of approximately 60%. To increase the sample size, a different method requiring less DNA input was applied at Genentech Laboratories, San Francisco, CA, USA. DNA was extracted from IHC-stained slides, amplified, and subsequently assessed using the DxS assay (DxS Ltd., Manchester, UK), which detects mutations E542K, E545K/D, and H1047R in exons 9 and 20. Mutational analysis per exon is presented to allow for pooling of the data on 328 cases overall. *PIK3CA* subgroups were defined based on the presence or absence of any mutation (mutant versus wild-type). “Wild-type” was only assigned to samples in which all reactions gave a valid result for “no mutation.” If one or more reactions failed, that sample was classified as “not assessable.”

Enzyme-linked immunosorbent assay (ELISA) was used to detect AREG, EGF, serum HER2 extracellular domain (sHER2), and transforming growth factor alpha (TGFα) in serum using an immunologic multiparametric chip technique (Roche Diagnostics). Levels of each serum marker were compared at baseline and at surgery in matched pairs from the same patients. Truncated forms of HER2 (the intracellular domain (ICD) and HER2 extracellular domain (ECD), where a ratio <1 indicates the presence of truncated forms), were measured in formalin-fixed, paraffin-embedded breast cancer tissue as described by Krüger et al. [20].

### Statistical and analytical methods

The patient population used (the biomarker population) was a subset of the intent-to-treat (ITT) population that contributed meaningful data to the analysis (Additional file 1: Table S1). In this analysis, median values were used as cutoffs for high and low biomarker levels for each of the continuous biomarkers, except for *PIK3CA* mutation and *c-Myc* amplification, where cutoffs were applied following a biologic rationale (wild-type versus mutant *PIK3CA* and *c-Myc*:centromere ratio ≥2). Instead of a data-driven cutoff, we used a prespecified median cutoff to reduce the number of false-positive findings due to the concomitant multiple testing and optimal cutoff selection. Box and whisker plots and summary statistics were used for initial data exploration and selection of relevant biomarkers. Spearman’s correlation was performed to identify the correlated biomarkers. Biomarkers with measurements near the limit of quantification were excluded from analyses.

False discovery rate was implemented to adjust for multiple comparisons at α = 0.05. Only groups A and B were considered for determining the additional benefit of adding pertuzumab to trastuzumab plus docetaxel.

Association between clinical variables (ER-positive versus ER-negative, local/inflammatory versus operable breast cancer, node-positive versus node-negative disease, and age ≥50 versus <50 years) and biomarkers were assessed in the overall biomarker population using the Chi-square test.

The association between biomarkers and pCR by treatment group were assessed by the Cochran–Mantel–Haenszel Chi-square test, stratified by hormone receptor status and breast cancer type. The treatment interaction test was performed for groups A and B to analyze the interaction between biomarkers and treatment in the association with pCR.

Analyses of the association between biomarkers and clinical variables, and of the association between biomarkers and pCR by treatment group, were repeated in ER-positive and ER-negative subgroups. These analyses were exploratory and secondary in nature, and therefore were not taken into account when adjusting for multiple comparisons.

## Results

### Overall population

#### Samples

The sample size per biomarker varied depending on technical success along with tissue quality and quantity (Additional file 1: Table S1). The biomarker group subset of each individual marker was fairly representative of the overall study population (data not shown). The levels of *AREG* and *Betacellulin* measured by qRT-PCR were generally very low (less than the limits of variability for these assays); hence, detailed interpretation of the data was not considered meaningful. The baseline levels of all biomarkers were well-balanced across all four treatment groups (Additional file 1: Table S2).

#### Association between median biomarker levels and pCR

**Fig. 1:**
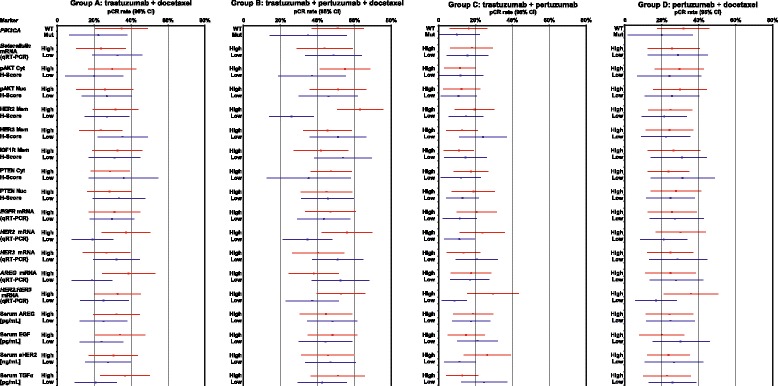
Relationship between biomarkers and pathologic complete response (*pCR*) by treatment group. *CR* concentration ratio, *cyt* cytoplasmic, *EGF* epidermal growth factor, *EGFR* epidermal growth factor receptor, *IGF1R* insulin-like growth factor 1 receptor, *Mem* membranous, *Mut* mutant, *Nuc* nuclear, *PIK3CA* gene encoding phosphoinositide 3-kinase catalytic subunit, *PTEN* phosphatase and tensin homolog, *qRT-PCR* quantitative reverse transcription polymerase chain reaction, *sHER2* serum HER2 extracellular domain, *TGF* transforming growth factor, *WT* wild-type

**Fig. 2:**
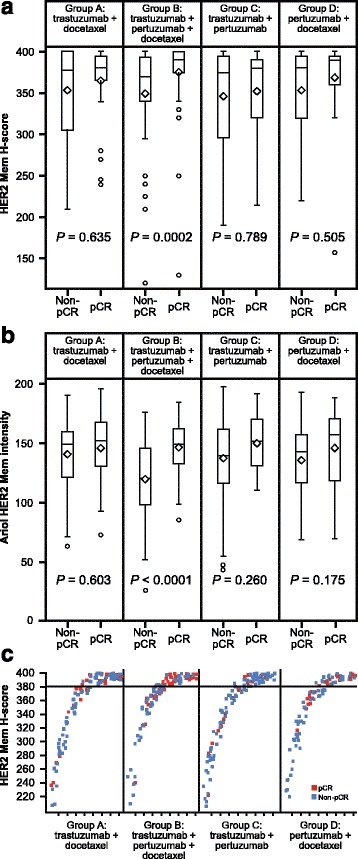
Human epidermal growth factor receptor 2 (*HER2*) membrane (*Mem*) analyses. **a** HER2 membrane scores versus outcome (pathologic complete response (*pCR*)) in all four treatment groups. **b** HER2 membrane staining intensity versus outcome in all four treatment groups (patients with missing pCR were excluded). **c** HER2 membrane score by outcome

In the overall biomarker population (Fig. 1 and Additional file 1: Table S3), high HER2 membrane protein expression, measured by modified H-score and stratified by ER status and breast cancer type, was associated with a significantly higher pCR rate in group B (pertuzumab, trastuzumab, and docetaxel) (*P* = 0.0002) (Fig. 2a). These findings were consistent with the assessment of HER2 when applying digital image analyses (Fig. 2b). In group B, higher HER2 levels, assessed by staining intensity score, were associated with higher pCR rates. Low serum TGFα was also associated with a significantly higher pCR rate in group C (*P* = 0.045) while the remaining biomarkers showed no significant difference when adjusted for ER status and breast cancer type (Additional file 1: Table S3).

**Fig. 3:**
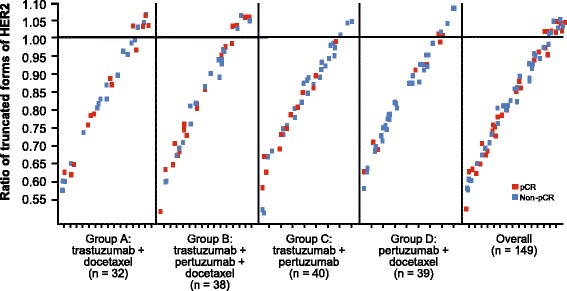
Human epidermal growth factor receptor 2 (*HER2*) extracellular domain (*ECD*)/intracellular domain (*ICD*) ratio. *pCR* pathologic complete response

In an exploratory analysis performed on truncated forms of HER2 without using high/low cutoffs, there was no association observed between pCR and these truncated proteins via the HER2 ECD/ICD ratio (Fig. 3).

#### Treatment interactions

When comparing group A with group B (Additional file 1: Table S4), only HER2 membrane protein expression had a significant interaction with treatment (*P* = 0.0236) with a large benefit from the combination of pertuzumab, trastuzumab, and docetaxel in the group with expression of HER2 above the median (odds ratio (OR) 2.07 (80% CI, 1.42 to 3.03, *P* = 0.01)) and no significant benefit for low HER2 expression. However, clustering of H-scores within a small dynamic range (median 380, maximum 400; Fig. 2c) limits the practical application of results.

#### *PIK3CA*

**Fig. 4:**
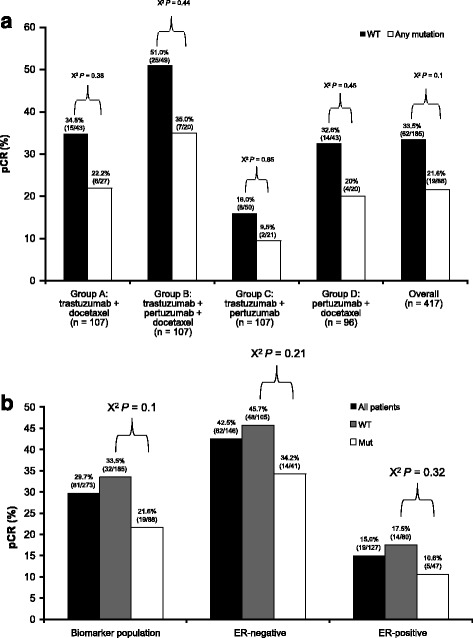
*PIK3CA* analyses. **a** *PIK3CA* status and relationship to outcome per treatment group. **b** Analysis of *PIK3CA* mutations by estrogen receptor (*ER*) status and relationship to outcome. *Mut* mutant, *pCR* pathologic complete response, *PIK3CA* gene encoding phosphoinositide 3-kinase, catalytic subunit, *WT* wild-type

In the overall population, *PIK3CA* mutations were found in 32% of all cases. A decreased pCR rate in patients carrying a *PIK3CA* mutation was seen consistently throughout all four groups, but this was not statistically significant in any of the groups or when the groups were pooled (Fig. 4a).

In a per-exon analysis, there was no trend of association between *PIK3CA* mutations and patterns of response to specific regimens (Table 1); therefore, the study groups were pooled for further analyses. Results showed that exon 9 mutations were associated with residual disease; a trend which was not seen for exon 20 mutations (Table 1). The number of cases was too small to interpret the results for exon 7.

**Table 1:** Analysis of *PIK3CA* mutations per exon and treatment group

	Group A		Group B		Group C		Group D		Overall (pooled groups)	
Exon *n* (Mut/total)	pCR, *n* (%)	non-pCR, *n* (%)	pCR, *n* (%)	non-pCR, *n* (%)	pCR, *n* (%)	non-pCR, *n* (%)	pCR, *n* (%)	non-pCR, *n* (%)	pCR, *n* (%)	non-pCR, *n* (%)
Exon 7	n = 2		n = 1		n = 1		n = 0		n = 4	
(4/290)	0	2 (100)	0	1 (100)	0	1 (100)	0	0	0	4 (100)
Exon 9	n = 8		n = 5		n = 5		n = 10		n = 28	
(28/328)	0	8 (100)	1 (20.0)	4 (80.0)	0	5 (100)	1 (10.0)	9 (90.0)	2 (7.1)	26 (92.9)
Exon 20	n = 20		n = 15		n = 17		n = 14		n = 66	
(66/338)	7 (35.0)	13 (65.0)	7 (46.7)	8 (53.3)	2 (11.8)	15 (88.2)	3 (21.4)	11 (78.6)	19 (28.8)	47 (71.2)

*Mut* mutant, *pCR* pathologic complete response, *PIK3CA* gene encoding phosphoinositide 3-kinase, catalytic subunit

#### Serum marker analyses

A comparison of baseline levels of the serum markers AREG, EGF, TGFα, and sHER2 between patients achieving pCR and those with residual disease did not predict pCR (*P* > 0.05 in all groups).

Changes in marker levels from baseline to post-neoadjuvant treatment were observed. Changes in EGF, TGFα, and sHER2 observed in the groups with pCR versus the groups with residual disease were comparable and did not allow us to discern a treatment benefit. Differences in AREG were observed in those achieving pCR versus those with residual disease but these were inconsistent across treatment groups.

There were no significant differences in the levels of EGF, TGFα, or sHER2 post-neoadjuvant treatment in the groups achieving pCR versus those with residual disease. Again AREG levels differed to some extent according to pCR status, but this was inconsistent between treatment groups.

**Fig. 5:**
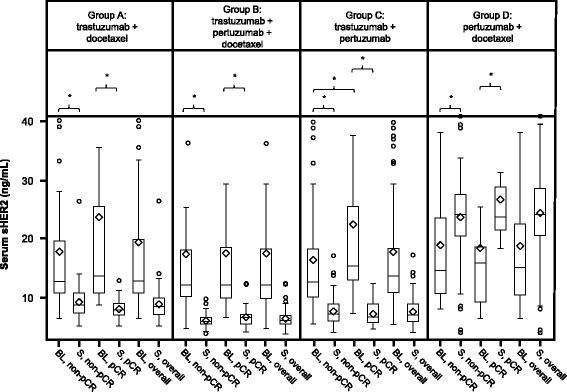
Matched pair analyses of serum human epidermal growth factor receptor 2 extracellular domain (*sHER2*) levels at baseline and at surgery (*S*). *P < 0.05 (exploratory analyses). *BL* baseline, *pCR* pathologic complete response

In general, the patterns of the serum markers were fairly consistent across treatment groups, with the exception of sHER2 (Fig. 5). In treatment groups A, B, and C the levels significantly decreased regardless of outcome. In treatment group D, which did not include trastuzumab, a significant increase was observed.

### ER-positive and ER-negative subgroups

As reported previously, ER status was correlated with pCR rates [8]. There were significantly different distributions of biomarker expression in the ER-positive versus ER-negative subgroups (false discovery rate <0.05). Membrane IGF1R protein expression, *HER3* mRNA expression, HER3 protein expression, cytoplasmic PTEN protein expression, and serum AREG were higher in ER-positive samples, while the *HER2*:*HER3* ratio (assessed by mRNA expression), *EGFR* mRNA expression, sHER2, *HER2* mRNA expression, and membrane HER2 protein expression were higher in ER-negative samples (Additional file 1: Table S5).

In general, the proportion of patients with *PIK3CA* mutations was comparable in the ER-positive and ER-negative subgroups. The subgroups were small, so it was not possible to investigate any imbalance on a per-exon basis, or on a potential correlation-to-outcome basis. The decreased benefit observed for patients carrying a *PIK3CA* mutation could not be attributed to an ER subgroup, as the benefit was comparable in ER-negative and ER-positive subgroups (Fig. 4b).

We investigated whether there was a different pattern of association for each biomarker with pCR according to ER status. For example, high *HER2* mRNA levels were associated with a higher pCR rate in group C (pertuzumab plus trastuzumab) with ER-negative tumors (*P* = 0.05) but not ER-positive tumors (*P* = 0.21). Detailed analyses of biomarker levels by ER status are available online (Additional file 2: Figure S1 and Additional file 1: Table S6).

**Fig. 6:**
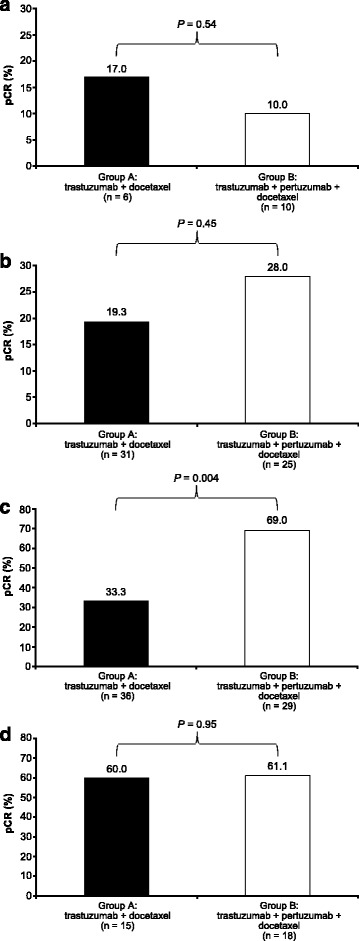
Insulin-like growth factor 1 receptor (*IGF1R*) expression according to estrogen receptor (ER) status. **a** Low IGF1R level/ER-positive group. **b** High IGF1R level/ER-positive group. **c** Low IGF1R level/ER-negative group. **d** High IGF1R level/ER-negative group. *pCR* pathologic complete response

Low levels of IGF1R were associated with higher pCR in group B (pertuzumab, trastuzumab, and docetaxel) versus group A (trastuzumab plus docetaxel) in the ER-negative (*P* = 0.004) but not the ER-positive subgroup (*P* = 0.54) (Fig. 6).

Higher levels of HER2 protein, cytoplasmic PTEN, sHER2, *EGFR* mRNA, *HER2* mRNA, and *HER2*/*HER3* mRNA were associated with ER-negative disease, while higher levels of HER3 protein, IGF1R protein, serum AREG, *AREG* mRNA, and *HER3* mRNA were associated with ER-positive disease. Other clinical variables were associated with higher levels of certain biomarkers; however, no clear biologic explanations were evident and multiplicity of testing may have introduced bias.

## Discussion

NeoSphere demonstrated that mandatory biomarker sampling is feasible in a clinical trial setting. Samples were available from nearly all patients and the planned panel of biomarkers could be assayed reliably in 99.8% (HER2) to 65.9% (*c-Myc*) of cases. While no new single marker could be confirmed as predictive of treatment benefit, these analyses revealed several important observations that will impact future trial designs.

Higher levels of HER2 membrane protein expression were associated with higher pCR rates with the combination of pertuzumab, trastuzumab, and docetaxel (group B) using both the modified H-score and digital image analyses. The digital readout resulted in a wider dynamic range compared with the manual scoring, showing a more pronounced effect in predicting pCR. While digital image analyses allowed us to better discern the pCR and non-pCR subgroups, HER2 levels remained highly overlapping between patients experiencing pCR versus patients not experiencing pCR and did not allow us to define clearly distinct populations that were either clinically or biologically meaningful.

The *PIK3CA* mutational status appears to have a relevant role in defining the likelihood of pCR with all treatment regimens tested in NeoSphere. However, while a decreased benefit was observed, the magnitude of the treatment effect with the addition of pertuzumab was maintained (a difference in pCR rates of approximately 15% in the pertuzumab, trastuzumab, and docetaxel group between patients with wild-type and mutant genes). This was similar to associations reported in the CLEOPATRA trial [21]. CLEOPATRA demonstrated shorter median progression-free survival with *PIK3CA* mutant tumors, regardless of treatment regimen [21]. Interestingly, in NeoSphere, only *PIK3CA* mutations in exon 9 were linked to a lack of sensitivity to HER2-directed monoclonal antibodies. Although independent confirmation is needed, due to the lower frequency and lack of statistical power for exon 9 mutations, this finding might be explained by the different mechanisms and downstream effects that are proposed based on preclinical observations for the gain of function mutations in helical (exon 9) versus kinase (exon 20) domain [22].

In CLEOPATRA, there was an association between poorer prognosis and pooled *PIK3CA* mutations at exons 9 and 20, although the benefit from pertuzumab was maintained regardless of mutation status [21]. Similar analyses from the TRYPHAENA study showed that pCR rates were lower, although not significantly, in patients with *PIK3CA* mutant tumors regardless of treatment regimen [23]. In addition, data from the EMILIA trial showed that patients with *PIK3CA* mutations had worse outcomes compared with those with wild-type tumors when treated with lapatinib plus capecitabine [24]. This was not the case with ado-trastuzumab emtansine, where the treatment benefit was similar for patients with wild-type and mutant tumors. This is an interesting finding and future trials should ascertain whether ado-trastuzumab emtansine can overcome the impact of *PIK3CA* mutation on outcome, either when given as monotherapy or in combination with other therapeutics such as PI3K inhibitors, with the goal of improving the poor prognosis of patients carrying *PIK3CA* mutations. The data from NeoSphere suggest that such an approach could be useful only in tumors carrying the activating exon 9 mutation.

Our results also provide clinical support for the blockade of HER2 cleavage by trastuzumab [5]. Trastuzumab is thought to inhibit HER2 shedding, as through binding to HER2 it may sterically hinder the metalloproteinases from accessing the cleavage sites [5]. By comparing serum marker levels at baseline and surgery, we showed that sHER2 levels decreased in all study groups except group D regardless of pCR status. Although serum HER2 extracellular domain did not predict pCR, this is a salient finding, as group D was the only group that did not include trastuzumab. To our knowledge this is the first report of clinical data to support this component of the proposed mechanism of action of trastuzumab. Although this is a reasonable mechanism based on our data, others cannot be ruled out.

Another relevant aspect that emerged from our analyses is the distinct difference between ER-positive and ER-negative tumors [15]. HER2-positive/ER-positive and HER2-positive/ER-negative breast cancer have different patterns of gene expression, and treatment outcome seems to be driven by different biologic pathways [11]. This effect was also seen in NeoSphere, where ER negativity was associated with increased likelihood of pCR with pertuzumab and trastuzumab [8], and in NOAH, where ER-/progesterone receptor- negativity was associated with increased benefit from trastuzumab [25, 26]. The different treatment effects according to ER status are reflected in a different molecular biomarker profile in ER-positive versus ER-negative tumors. At this point our data do not provide information that would result in patients receiving altered treatment, but future trial designs should take into account the intrinsic differences in marker profiles and related sensitivity to treatment.

A limitation of the biomarker analysis was that there was a narrow range of HER2 protein levels with little numerical difference between cutoffs, hindering an appropriate exploration of HER2 protein-related impact on treatment outcome. In addition, there was no significant difference in the pCR rates of patients in the subgroups with higher and lower HER2 levels, who received pertuzumab plus trastuzumab without docetaxel (group C). As the chemotherapy backbone is not known to impact HER2-related effects, the association between HER2 protein levels and pCR rates should have been detectable in both of the groups receiving trastuzumab plus pertuzumab.

Given that we were limited by small numbers of patients in some subpopulations, our analyses did not provide any additional predictive markers supporting a refinement of the HER2-positive target population treated with trastuzumab, pertuzumab, and docetaxel. It should be acknowledged that the use of the median cutoff for biomarker assessment could have overlooked a predictive value of some of these biomarkers at different, biologically meaningful, cutoffs that have not been explored in this study. However, our analysis did highlight the different biology of ER-positive and ER-negative HER2-positive tumors. The observation of a lower likelihood of pCR with trastuzumab, pertuzumab, and docetaxel in tumors harboring *PIK3CA* mutation at exon 9 is limited by the small sample size and warrants further exploration in larger datasets.

## Conclusions

In conclusion, our data show that conventional assessment of HER2 by IHC or FISH should continue at this time, and that HER2 remains the only biomarker suitable for patient and/or regimen selection in this population. The observations in NeoSphere, however, may guide future neoadjuvant trial designs.

## Additional files

Additional file 1: Table S1. Biomarker analyses on the intent-to-treat population: sample sizes and technical success rates. *As a percentage of the ITT population (N = 417). *CR* concentration ratio, *Cyt* cytoplasmic, EGF epidermal growth factor, *EGFR* epidermal growth factor receptor, *ELISA* enzyme-linked immunosorbent assay, *FISH* fluorescence *in situ* hybridization, *IGF1R* insulin-like growth factor 1 receptor, *IHC* immunohistochemistry, *Mem* membranous, *Nuc* nuclear, *PIK3CA* gene encoding phosphoinositide 3-kinase catalytic subunit, *PTEN* phosphatase and tensin homolog, *qRT-PCR* quantitative reverse transcription PCR, *sHER2* serum HER2 extracellular domain, *SNP* single nucleotide polymorphism, *TGF* transforming growth factor. **Table S2.** Baseline levels of all biomarkers in all four treatment groups (biomarker population, based on the intent-to-treat population). *CR* concentration ratio, *Cyt* cytoplasmic, EGF epidermal growth factor, *EGFR* epidermal growth factor receptor, *ELISA* enzyme-linked immunosorbent assay, *FISH* fluorescence *in situ* hybridization, *IGF1R* insulin-like growth factor 1 receptor, *IHC* immunohistochemistry, *Mem* membranous, *Nuc* nuclear, *PIK3CA* gene encoding phosphoinositide 3-kinase catalytic subunit, *PTEN* phosphatase and tensin homolog, *qRT-PCR* quantitative reverse transcription PCR, *sHER2* serum HER2 extracellular domain, *SNP* single nucleotide polymorphism, *TGF* transforming growth factor. **Table S3.** Relationship between biomarkers and pCR, adjusted for hormone receptor status and breast cancer type (biomarker population, based on the intent-to-treat population). *CR* concentration ratio, *Cyt* cytoplasmic, EGF epidermal growth factor, *EGFR* epidermal growth factor receptor, *ELISA* enzyme-linked immunosorbent assay, *FISH* fluorescence *in situ* hybridization, *IGF1R* insulin-like growth factor 1 receptor, *IHC* immunohistochemistry, *Mem* membranous, *Mut *mutant﻿, *Nuc* nuclear, *pCR* pathologic complete response, *PIK3CA* gene encoding phosphoinositide 3-kinase catalytic subunit, *PTEN* phosphatase and tensin homolog, *qRT-PCR* quantitative reverse transcription PCR, *sHER2* serum HER2 extracellular domain, *SNP* single nucleotide polymorphism, *TGF* transforming growth factor, *WT* wild-type. Cochran–Mantel–Haenszel Chi-square test based on biomarker subgroup x pCR status (2 × 2) stratified by hormone receptor status and breast cancer type. **Table S4.** Treatment and biomarker interaction tests comparing groups A (trastuzumab plus docetaxel) and B (pertuzumab, trastuzumab, and docetaxel) (median cut-point; biomarker population, based on the intent-to-treat population). *Subgroup defined by using a cutoff for target:centromere ratio of 2. ^†^Subgroup defined using mutation versus no mutation. *CR* concentration ratio, *Cyt* cytoplasmic, EGF epidermal growth factor, *EGFR* epidermal growth factor receptor, *ELISA* enzyme-linked immunosorbent assay, *FISH* fluorescence *in situ* hybridization, *IGF1R* insulin-like growth factor 1 receptor, *IHC* immunohistochemistry, *Mem* membranous, *Nuc* nuclear, *pCR* pathologic complete response, *PIK3CA* gene encoding phosphoinositide 3-kinase catalytic subunit, *PTEN* phosphatase and tensin homolog, *qRT-PCR* quantitative reverse transcription PCR, *SNP* single nucleotide polymorphism, *TGF* transforming growth factor. **Table S5.** Relationship between biomarkers and pCR by hormone receptor status (biomarker population, based on the intent-to-treat population). *CR* concentration ratio, *Cyt* cytoplasmic, EGF epidermal growth factor, *EGFR* epidermal growth factor receptor, *ELISA* enzyme-linked immunosorbent assay, *FISH* fluorescence *in situ* hybridization, *IGF1R* insulin-like growth factor 1 receptor, *IHC* immunohistochemistry, *Mem* membranous, *Nuc* nuclear, *pCR* pathologic complete response, *PIK3CA* gene encoding phosphoinositide 3-kinase catalytic subunit, *PTEN* phosphatase and tensin homolog, *qRT-PCR* quantitative reverse transcription PCR, *sHER2* serum HER2 extracellular domain, *TGF* transforming growth factor. **Table S6.** Detailed analyses of biomarker levels by ER status. *CR* concentration ratio, *Cyt* cytoplasmic, EGF epidermal growth factor, *EGFR* epidermal growth factor receptor, *ELISA* enzyme-linked immunosorbent assay, *FISH* fluorescence *in situ* hybridization, *IGF1R* insulin-like growth factor 1 receptor, *IHC* immunohistochemistry, *Mem* membranous, *Nuc* nuclear, *pCR* pathologic complete response, *PIK3CA* gene encoding phosphoinositide 3-kinase, catalytic subunit; *PTEN* phosphatase and tensin homolog, *qRT-PCR* quantitative reverse transcription PCR, *sHER2* serum HER2 extracellular domain, *TGF* transforming growth factor; *WT* wild-type. Cochran–Mantel–Haenszel Chi-square test based on biomarker subgroup × pCR status (2 × 2) stratified by breast cancer type. (DOCX 59 kb)
Additional file 2:
**Figure S1.** Relationship between biomarkers and pCR by treatment group and estrogen receptor status. *CR* concentration ratio, *Cyt* cytoplasmic, EGF epidermal growth factor, *EGFR* epidermal growth factor receptor, *ELISA* enzyme-linked immunosorbent assay, *FISH* fluorescence *in situ* hybridization, *IGF1R* insulin-like growth factor 1 receptor, *IHC* immunohistochemistry, *Mem* membranous, *Nuc* nuclear, *pCR* pathologic complete response, *PIK3CA* gene encoding phosphoinositide 3-kinase, catalytic subunit; *PTEN* phosphatase and tensin homolog, *qRT-PCR* quantitative reverse transcription PCR, *sHER2* serum HER2 extracellular domain, *TGF* transforming growth factor; *WT* wild-type. (PDF 836 kb)

## Abbreviations

ADCC: Antibody-dependent cell-mediated cytotoxicity
AREG: Amphiregulin
CR: Concentration ratio
cyt: Cytoplasmic
ECD: Extracellular domain
EGF: Epidermal growth factor
EGFR: Epidermal growth factor receptor
ELISA: Enzyme-linked immunosorbent assay
ER: Estrogen receptor
FISH: Fluorescence *in situ* hybridization
HER2: Human epidermal growth factor receptor 2
HER3: Human epidermal growth factor receptor 3
ICD: Intracellular domain
IGF1R: Insulin-like growth factor 1 receptor
IHC: Immunohistochemistry
Mem: Membranous
Mut: Mutant
Nuc: Nuclear
pCR: Pathologic complete response
PIK3CA: Gene encoding phosphoinositide 3-kinase, catalytic subunit
PTEN: Phosphatase and tensin homolog
qRT-PCR: Quantitative reverse transcription polymerase chain reaction
sHER2: Serum human epidermal growth factor receptor 2 extracellular domain
SNP: Single nucleotide polymorphism
TGFα: Transforming growth factor alpha
WT: Wild-type
