# Supplementary material for: Biomarker analysis of the NeoSphere study: pertuzumab, trastuzumab, and docetaxel versus trastuzumab plus docetaxel, pertuzumab plus trastuzumab, or pertuzumab plus docetaxel for the neoadjuvant treatment of HER2-positive breast cancer
Source: Breast Cancer Res. 2017 Feb 9;19:16. doi: 10.1186/s13058-017-0806-9 (PMC5299741; doi:10.1186/s13058-017-0806-9)
Supplement: Additional file 1: Table S1. — Biomarker analyses on the intent-to-treat population: sample sizes and technical success rates. *As a percentage of the ITT population (N = 417). CR concentration ratio, Cyt cytoplasmic, EGF epidermal growth factor, EGFR epidermal growth factor receptor, ELISA enzyme-linked immunosorbent assay, FISH fluorescence in situ hybridization, IGF1R insulin-like growth factor 1 receptor, IHC immunohistochemistry, Mem membranous, Nuc nuclear, PIK3CA gene encoding phosphoinositide 3-kinase catalytic subunit, PTEN phosphatase and tensin homolog, qRT-PCR quantitative reverse transcription PCR, sHER2 serum HER2 extracellular domain, SNP single nucleotide polymorphism, TGF transforming growth factor. Table S2. Baseline levels of all biomarkers in all four treatment groups (biomarker population, based on the intent-to-treat population). CR concentration ratio, Cyt cytoplasmic, EGF epidermal growth factor, EGFR epidermal growth factor receptor, ELISA enzyme-linked immunosorbent assay, FISH fluorescence in situ hybridization, IGF1R insulin-like growth factor 1 receptor, IHC immunohistochemistry, Mem membranous, Nuc nuclear, PIK3CA gene encoding phosphoinositide 3-kinase catalytic subunit, PTEN phosphatase and tensin homolog, qRT-PCR quantitative reverse transcription PCR, sHER2 serum HER2 extracellular domain, SNP single nucleotide polymorphism, TGF transforming growth factor. Table S3. Relationship between biomarkers and pCR, adjusted for hormone receptor status and breast cancer type (biomarker population, based on the intent-to-treat population). CR concentration ratio, Cyt cytoplasmic, EGF epidermal growth factor, EGFR epidermal growth factor receptor, ELISA enzyme-linked immunosorbent assay, FISH fluorescence in situ hybridization, IGF1R insulin-like growth factor 1 receptor, IHC immunohistochemistry, Mem membranous, Mut mutant﻿, Nuc nuclear, pCR pathologic complete response, PIK3CA gene encoding phosphoinositide 3-kinase catalytic subunit, PTEN phosphatase and tensin homolog, q [file 13058_2017_806_MOESM1_ESM.docx]

Table S1. Biomarker analyses on the intent-to-treat population: sample sizes and technical success rates.

| **Assay method** | **Biomarker** | **Sample size,**  **n (%)*** |
| --- | --- | --- |
| IHC | HER2 Mem H-score | 416 (99.8) |
|  | HER3 Mem H-score | 377 (90.4) |
|  | IGF1R Mem H-score | 339 (81.3) |
|  | PTEN Cyt H-score | 373 (89.4) |
|  | PTEN Nuc H-score | 373 (89.4) |
|  | pAKT Cyt H-score | 299 (71.7) |
|  | pAKT Nuc H-score | 299 (71.7) |
|  | HER2 ECD/ICD ratio | 163 (39.1) |
| qRT-PCR | *Amphiregulin* CR | 358 (85.9) |
|  | *Betacellulin* CR | 324 (77.7) |
|  | *EGFR* CR | 377 (90.4) |
|  | *HER2* CR | 387 (92.8) |
|  | *HER2*/*HER3* CR | 384 (92.1) |
|  | *HER3* CR | 384 (92.1) |
| FISH* | *c-Myc* (target:centromere ratio) | 275 (65.9) |
| ELISA – serum | Amphiregulin (pg/mL) | 384 (92.1) |
|  | EGF (pg/mL) | 384 (92.1) |
|  | TGF-alpha (pg/mL) | 384 (92.1) |
|  | sHER2 (ng/mL) | 381 (91.4) |
| SNP | *PIK3CA* (any mutation) | 328 (78.7) |

* As a percentage of the ITT population (N = 417).

CR, concentration ratio; Cyt, cytoplasmic; EGF, epidermal growth factor; EGFR, epidermal growth factor receptor; ELISA, enzyme-linked immunosorbent assay; FISH, fluorescence *in situ* hybridization; IGF1R, insulin-like growth factor 1 receptor; IHC, immunohistochemistry; Mem, membranous; Nuc, nuclear; *PIK3CA*, gene encoding phosphoinositide 3-kinase, catalytic subunit; PTEN, phosphatase and tensin homolog; qRT-PCR, quantitative reverse transcription PCR; sHER2, serum HER2 extracellular domain; SNP, single nucleotide polymorphism; TGF, transforming growth factor.

Table S2. Baseline levels of all biomarkers in all four treatment groups (biomarker population, based on the intent-to-treat population).

|  | **Overall population** | **Group A: trastuzumab + docetaxel** | **Group B: trastuzumab + pertuzumab + docetaxel** | **Group C: trastuzumab + pertuzumab** | **Group D: pertuzumab + docetaxel** |
| --- | --- | --- | --- | --- | --- |
|  | **N = 417** | **N = 107** | **N = 107** | **N = 107** | **N = 96** |
| **IHC** |  |  |  |  |  |
| HER2 Mem H‑score |  |  |  |  |  |
| n | 416 | 106 | 107 | 107 | 96 |
| Median | 380.0 | 380.0 | 380.0 | 375.0 | 380.0 |
| Range | 130.0 to 400.0 | 210.0 to 400.0 | 130.0 to 400.0 | 190.0 to 400.0 | 157.0 to 400.0 |
| HER3 Mem H‑score |  |  |  |  |  |
| n | 377 | 99 | 96 | 99 | 83 |
| Median | 40.00 | 40.00 | 57.50 | 50.00 | 40.00 |
| Range | 0.000 to 390.0 | 0.000 to 330.0 | 0.000 to 310.0 | 0.000 to 300.0 | 0.000 to 390.0 |
| pAKT Cyt  H-score |  |  |  |  |  |
| n | 299 | 72 | 78 | 79 | 70 |
| Median | 200.0 | 200.0 | 200.0 | 200.0 | 200.0 |
| Range | 0.000 to 400.0 | 0.000 to 400.0 | 0.000 to 300.0 | 0.000 to 350.0 | 0.000 to 300.0 |
| IGF1R Mem H-score |  |  |  |  |  |
| n | 339 | 88 | 82 | 91 | 78 |
| Median | 20.00 | 20.00 | 20.00 | 40.00 | 20.00 |
| Range | 0.000 to 400.0 | 0.000 to 393.0 | 0.000 to 300.0 | 0.000 to 390.0 | 0.000 to 400.0 |
| PTEN Cyt  H-score |  |  |  |  |  |
| n | 373 | 95 | 95 | 98 | 85 |
| Median | 200.0 | 200.0 | 225.0 | 200.0 | 200.0 |
| Range | 0.000 to 400.0 | 0.000 to 400.0 | 0.000 to 390.0 | 0.000 to 400.0 | 0.000 to 370.0 |
| pAKT Nuc  H-score |  |  |  |  |  |
| n | 299 | 72 | 78 | 79 | 70 |
| Median | 160.0 | 120.0 | 160.0 | 160.0 | 167.5 |
| Range | 0.000 to 390.0 | 0.000 to 390.0 | 0.000 to 300.0 | 0.000 to 360.0 | 0.000 to 330.0 |
| PTEN Nuc  H-score |  |  |  |  |  |
| n | 373 | 95 | 95 | 98 | 85 |
| Median | 145.0 | 160.0 | 150.0 | 120.0 | 140.0 |
| Range | 0.000 to 400.0 | 0.000 to 400.0 | 0.000 to 310.0 | 0.000 to 400.0 | 0.000 to 316.0 |
| **Serum** |  |  |  |  |  |
| Amphiregulin (pg/mL) |  |  |  |  |  |
| n | 384 | 97 | 99 | 101 | 87 |
| Median | 4.800 | 5.200 | 4.400 | 4.600 | 4.900 |
| Range | 1.600 to 82.20 | 2.000 to 81.60 | 1.700 to 82.20 | 2.100 to 42.60 | 1.6 to 49.70 |
| EGF (pg/mL) |  |  |  |  |  |
| n | 384 | 97 | 99 | 101 | 87 |
| Median | 229.3 | 227.9 | 264.9 | 218.4 | 224.5 |
| Range | 2.800 to 1591 | 17.40 to 1526 | 2.800 to 1330 | 12.40 to 1270 | 17.20 to 1591 |
| TGFα (pg/mL) |  |  |  |  |  |
| n | 384 | 97 | 99 | 101 | 87 |
| Median | 14.35 | 14.40 | 13.40 | 15.10 | 14.10 |
| Range | 1.800 to 80.50 | 1.800 to 80.50 | 2.800 to 72.00 | 1.800 to 58.50 | 3.600 to 76.40 |
| **qRT-PCR** |  |  |  |  |  |
| *Amphiregulin* CR |  |  |  |  |  |
| n | 358 | 92 | 90 | 94 | 82 |
| Median | 0.056 | 0.051 | 0.066 | 0.054 | 0.051 |
| Range | 0.000 to 18.80 | 0.000 to 18.80 | 0.001 to 1.470 | 0.000 to 3.160 | 0.000 to 2.000 |
| *Betacellulin* CR |  |  |  |  |  |
| n | 324 | 84 | 82 | 84 | 74 |
| Median | 0.010 | 0.010 | 0.011 | 0.012 | 0.011 |
| Range | 0.000 to 0.314 | 0.000 to 0.166 | 0.000 to 0.314 | 0.000 to 0.222 | 0.001 to 0.099 |
| *EGFR* CR |  |  |  |  |  |
| n | 377 | 96 | 97 | 99 | 85 |
| Median | 0.135 | 0.101 | 0.137 | 0.144 | 0.133 |
| Range | 0.005 to 9.990 | 0.005 to 1.010 | 0.008 to 1.260 | 0.005 to 9.990 | 0.008 to 1.640 |
| *HER2* CR |  |  |  |  |  |
| n | 387 | 100 | 99 | 102 | 86 |
| Median | 13.30 | 14.00 | 13.70 | 12.40 | 15.45 |
| Range | 0.203 to 108.0 | 0.210 to 70.00 | 0.763 to 65.80 | 0.248 to 108.0 | 0.203 to 108.0 |
| *HER2/HER3* CR |  |  |  |  |  |
| n | 384 | 98 | 98 | 102 | 86 |
| Median | 29.20 | 32.10 | 33.45 | 24.35 | 26.55 |
| Range | 0.697 to 260.0 | 0.933 to 252.0 | 0.697 to 165.0 | 1.210 to 260.0 | 1.780 to 215.0 |
| *HER3* CR |  |  |  |  |  |
| n | 384 | 98 | 98 | 102 | 86 |
| Median | 0.505 | 0.446 | 0.488 | 0.523 | 0.534 |
| Range | 0.017 to 4.110 | 0.017 to 3.360 | 0.033 to 2.660 | 0.052 to 3.780 | 0.035 to 4.110 |
| **FISH** |  |  |  |  |  |
| Target:centromere ratio (*c-Myc*) |  |  |  |  |  |
| n | 275 | 74 | 68 | 67 | 66 |
| Median | 1.470 | 1.440 | 1.355 | 1.634 | 1.485 |
| Range | 0.780 to 10.66 | 0.780 to 10.66 | 1.030 to 9.250 | 1.020 to 10.56 | 0.960 to 9.050 |

CR, concentration ratio; Cyt, cytoplasmic; EGF, epidermal growth factor; EGFR, epidermal growth factor receptor; ELISA, enzyme-linked immunosorbent assay; FISH, fluorescence *in situ* hybridization; IGF1R, insulin-like growth factor 1 receptor; IHC, immunohistochemistry; Mem, membranous; Nuc, nuclear; *PIK3CA*, gene encoding phosphoinositide 3-kinase, catalytic subunit; PTEN, phosphatase and tensin homolog; qRT-PCR, quantitative reverse transcription polymerase chain reaction; TGF, transforming growth factor.

Table S3. Relationship between biomarkers and pCR, adjusted for hormone receptor status and breast cancer type (biomarker population, based on the intent-to-treat population).

|  | **Group A: trastuzumab + docetaxel** | **Group B: trastuzumab + pertuzumab + docetaxel** | **Group C: trastuzumab + pertuzumab** | **Group D: pertuzumab + docetaxel** |
| --- | --- | --- | --- | --- |
|  | **N = 107** | **N = 107** | **N = 107** | **N = 96** |
| **IHC** |  |  |  |  |
| HER2 Mem H‑score |  |  |  |  |
| High | 17/54 (31.48%) | 36/57 (63.13%) | 10/52 (19.23%) | 13/51 (25.49%) |
| Low | 14/52 (26.92%) | 13/50 (26.00%) | 8/55 (14.55%) | 10/45 (22.22%) |
| Chi-square | 0.8899 | 0.0010 | 0.6437 | 0.9164 |
| HER3 Mem H‑score |  |  |  |  |
| High | 12/51 (23.53%) | 25/55 (45.45%) | 7/57 (12.28%) | 11/44 (25.00%) |
| Low | 17/48 (35.42%) | 21/41 (51.22%) | 10/42 (23.81%) | 9/39 (23.08%) |
| Chi-square | 0.4079 | 0.5983 | 0.4863 | 0.9418 |
| pAKT Cyt  H-score |  |  |  |  |
| High | 14/47 (29.79%) | 28/51 (54.90%) | 6/53 (11.32%) | 14/46 (30.43%) |
| Low | 5/25 (20.00%) | 10/27 (37.04%) | 3/26 (11.54%) | 6/24 (25.00%) |
| Chi-square | 0.3942 | 0.3008 | 1.0000 | 0.6058 |
| IGF1R Mem H-score |  |  |  |  |
| High | 15/46 (32.61%) | 18/43 (41.86%) | 6/56 (10.71%) | 11/41 (26.83%) |
| Low | 13/42 (30.95%) | 21/39 (53.85%) | 5/35 (14.29%) | 11/37 (29.73%) |
| Chi-square | 0.3417 | 0.9932 | 0.5085 | 0.7610 |
| PTEN Cyt  H-score |  |  |  |  |
| High | 20/70 (28.57%) | 37/78 (47.44%) | 11/64 (17.19%) | 14/57 (24.56%) |
| Low | 9/25 (36.00%) | 6/17 (35.29%) | 4/34 (11.76%) | 9/28 (32.14%) |
| Chi-square | 0.6050 | 0.0996 | 0.2080 | 0.8134 |
| pAKT Nuc  H-score |  |  |  |  |
| High | 8/31 (25.81%) | 21/41 (51.22%) | 5/41 (12.20%) | 12/39 (30.77%) |
| Low | 11/41 (26.83%) | 17/37 (45.95%) | 4/38 (10.53%) | 8/31 (25.81%) |
| Chi-square | 0.7296 | 0.4140 | 0.5823 | 0.5325 |
| PTEN Nuc  H-score |  |  |  |  |
| High | 15/53 (28.30%) | 22/49 (44.90%) | 8/43 (18.60%) | 12/42 (28.57%) |
| Low | 14/42 (33.33%) | 21/46 (45.65%) | 7/55 (12.73%) | 11/43 (25.58%) |
| Chi-square | 0.4364 | 0.8855 | 0.1565 | 0.5919 |
| **Serum** |  |  |  |  |
| Amphiregulin (pg/mL) |  |  |  |  |
| High | 17/53 (32.08%) | 21/47 (44.68%) | 9/49 (18.37%) | 11/44 (25.00%) |
| Low | 11/44 (25.00%) | 25/52 (48.08%) | 9/52 (17.31%) | 11/43 (25.58%) |
| Chi-square | 0.3249 | 0.7598 | 0.8756 | 0.6483 |
| EGF (pg/mL) |  |  |  |  |
| High | 16/47 (34.04%) | 26/54 (48.15%) | 7/48 (14.58%) | 9/43 (20.93%) |
| Low | 12/50 (24.00%) | 20/45 (44.44%) | 11/53 (20.75%) | 13/44 (29.55%) |
| Chi-square | 0.1966 | 0.5762 | 0.4493 | 0.5319 |
| TGFα (pg/mL) |  |  |  |  |
| High | 18/49 (36.73%) | 23/45 (51.11%) | 7/56 (12.50%) | 10/42 (23.81%) |
| Low | 10/48 (20.83%) | 23/54 (42.59%) | 11/45 (24.44%) | 12/45 (26.67%) |
| Chi-square | 0.0642 | 0.4000 | 0.0445 | 0.8828 |
| **qRT-PCR** |  |  |  |  |
| *EGFR* CR |  |  |  |  |
| High | 13/42 (30.95%) | 24/51 (47.06%) | 11/54 (20.37%) | 11/42 (26.19%) |
| Low | 16/54 (29.63%) | 20/46 (43.48%) | 5/45 (11.11%) | 12/43 (27.91%) |
| Chi-square | 0.9919 | 0.5121 | 0.3459 | 0.9318 |
| *HER2* CR |  |  |  |  |
| High | 20/53 (37.74%) | 28/50 (56.00%) | 11/47 (23.40%) | 14/45 (31.11%) |
| Low | 9/47 (19.15%) | 17/49 (34.69%) | 6/55 (10.91%) | 9/41 (21.95%) |
| Chi-square | 0.0774 | 0.1616 | 0.2009 | 0.3937 |
| *HER2/HER3* CR |  |  |  |  |
| High | 18/55 (32.73%) | 29/55 (52.73%) | 12/41 (29.27%) | 15/41 (36.59%) |
| Low | 11/43 (25.58%) | 16/43 (37.21%) | 5/61 (8.20%) | 8/45 (17.78%) |
| Chi-square | 0.9616 | 0.5524 | 0.0790 | 0.1765 |
| *HER3* CR |  |  |  |  |
|  |  |  |  |  |
| High | 12/45 (26.67%) | 19/47 (40.43%) | 7/53 (13.21%) | 12/47 (25.53%) |
| Low | 17/53 (32.08%) | 26/51 (50.98%) | 10/49 (20.41%) | 11/39 (28.21%) |
| Chi-square | 0.9141 | 0.5874 | 0.8707 | 0.7987 |
| **FISH** |  |  |  |  |
| Target:centromere ratio (*c-Myc*) |  |  |  |  |
| High | 6/19 (31.58%) | 12/24 (50.00%) | 2/23 (8.70%) | 3/21 (14.29%) |
| Low | 19/55 (34.55%) | 19/44 (43.18%) | 6/44 (13.64%) | 13/45 (28.89%) |
| Chi-square | 0.8257 | 0.4717 | 0.4630 | 0.1566 |
| **SNP** |  |  |  |  |
| *PIK3CA*  (any mutation) |  |  |  |  |
| WT | 15/43 (34.88%) | 25/49 (51.02%) | 8/50 (16.00%) | 14/43 (32.56%) |
| Mut | 6/27 (22.22%) | 7/20 (35.00%) | 2/21 (9.52%) | 4/20 (20.00%) |
| Chi-square | 0.37833 | 0.43893 | 0.65016 | 0.45080 |

CR, concentration ratio; Cyt, cytoplasmic; EGF, epidermal growth factor; EGFR, epidermal growth factor receptor; ELISA, enzyme-linked immunosorbent assay; FISH, fluorescence *in situ* hybridization; IGF1R, insulin-like growth factor 1 receptor; IHC, immunohistochemistry; Mem, membranous; Mut, mutant; Nuc, nuclear; pCR, pathologic complete response; *PIK3CA*, gene encoding phosphoinositide 3-kinase, catalytic subunit; PTEN, phosphatase and tensin homolog; qRT-PCR, quantitative reverse transcription polymerase chain reaction; sHER2, serum HER2 extracellular domain; SNP, single nucleotide polymorphism; TGF, transforming growth factor; MT, wild-type.

Cochran–Mantel–Haenszel Chi-square test based on biomarker subgroup x pCR status (2x2) stratified by hormone receptor status and breast cancer type.

Table S4. Treatment and biomarker interaction tests comparing groups A (trastuzumab plus docetaxel) and B (pertuzumab, trastuzumab, and docetaxel) (median cut-point; biomarker population, based on the intent-to-treat population).

|  |  | **Treatment* biomarker interaction** | | |
| --- | --- | --- | --- | --- |
|  |  | **pCR, %** | |  |
| **Assay method** | **Biomarker** | **Group A: trastuzumab**  **+ docetaxel** | **Group B:**  **pertuzumab + trastuzumab + docetaxel** | ***P* value** |
| IHC | HER2 Mem H-score  High  Low | 31.48  26.92 | 63.16  26.00 | 0.0236 |
|  | HER3 Mem H-score  High  Low | 23.53  35.42 | 45.45  51.22 | 0.5691 |
|  | IGF1R Mem H-score  High  Low | 32.61  30.95 | 41.86  53.85 | 0.3821 |
|  | PTEN Cyt H-score  High  Low | 28.57  36.00 | 47.44  35.29 | 0.2561 |
|  | PTEN Nuc H-score  High  Low | 28.30  33.33 | 44.90  45.65 | 0.7352 |
|  | pAKT Cyt H-score  High  Low | 29.79  20.00 | 54.90  37.04 | 0.796 |
|  | pAKT Nuc H-score  High  Low | 25.81  26.83 | 51.22  45.95 | 0.7086 |
| qRT-PCR | *EGFR* CR  High  Low | 30.95  29.63 | 47.06  43.48 | 0.8924 |
|  | *HER2* CR  High  Low | 37.74  19.15 | 56.00  34.69 | 0.9159 |
|  | *HER2/HER3* CR  High  Low | 32.73  25.58 | 52.73  37.21 | 0.6424 |
|  | *HER3* CR  High  Low | 26.67  32.08 | 40.43  50.98 | 0.784 |
| FISH* | *c-Myc* (target:centromere ratio)  High  Low | 31.58  34.55 | 50.00  43.18 | 0.5927 |
| ELISA – serum | Amphiregulin (pg/mL)  High  Low | 32.08  25.00 | 44.68  48.08 | 0.4259 |
|  | EGF (pg/mL)  High  Low | 34.04  24.00 | 48.15  44.44 | 0.5729 |
|  | TGF-alpha (pg/mL)  High  Low | 36.73  20.83 | 51.11  42.59 | 0.4662 |

* Subgroup defined by using a cutoff for target:centromere ratio of 2.

^†^ Subgroup defined using mutation versus no mutation.

CR, concentration ratio; Cyt, cytoplasmic; EGF, epidermal growth factor; EGFR, epidermal growth factor receptor; ELISA, enzyme-linked immunosorbent assay; FISH, fluorescence *in situ* hybridization; IGF1R, insulin-like growth factor 1 receptor; IHC, immunohistochemistry; Mem, membranous; Nuc, nuclear; pCR, pathologic complete response; PTEN, phosphatase and tensin homolog; qRT-PCR, quantitative reverse transcription polymerase chain reaction; SNP, single nucleotide polymorphism; TGF, transforming growth factor.

Table S5. Relationship between biomarkers and pCR by hormone receptor status (biomarker population, based on the intent-to-treat population).

| **Assay method** | **Biomarker** | **FDR *P* value ER-positive versus ER-negative** |
| --- | --- | --- |
| IHC | HER2 Mem H-score | < 0.00001 |
|  | HER3 Mem H-score | 0.00078 |
|  | IGF1R Mem H-score | < 0.00001 |
|  | PTEN Cyt H-score | < 0.00001 |
|  | PTEN Nuc H-score | 0.33813 |
|  | pAKT Cyt H-score | 0.39162 |
|  | pAKT Nuc H-score | 0.46052 |
| qRT-PCR | *EGFR* CR | 0.01221 |
|  | *HER2* CR | 0.01450 |
|  | *HER2*/*HER3* CR | < 0.00001 |
|  | *HER3* CR | < 0.00001 |
| FISH* | *c-Myc* (target:centromere ratio) | 0.39162 |
| ELISA – serum | Amphiregulin (pg/mL) | 0.02087 |
|  | EGF (pg/mL) | 0.39162 |
|  | TGF-alpha (pg/mL) | 0.33813 |
|  | sHER2 (ng/mL) | 0.00017 |

CR, concentration ratio; Cyt, cytoplasmic; EGF, epidermal growth factor; EGFR, epidermal growth factor receptor; ELISA, enzyme-linked immunosorbent assay; FISH, fluorescence *in situ* hybridization; IGF1R, insulin-like growth factor 1 receptor; IHC, immunohistochemistry; Mem, membranous; Nuc, nuclear; pCR, pathologic complete response; PTEN, phosphatase and tensin homolog; qRT-PCR, quantitative reverse transcription polymerase chain reaction; sHER2, serum HER2 extracellular domain; TGF, transforming growth factor.

Table S6. Detailed analyses of biomarker levels by ER status.

|  |  | **Group A: trastuzumab + docetaxel** | **Group B: trastuzumab + pertuzumab + docetaxel** | **Group C: trastuzumab + pertuzumab** | **Group D: pertuzumab + docetaxel** |
| --- | --- | --- | --- | --- | --- |
|  |  | **N = 107** | **N = 107** | **N = 106** | **N = 96** |
| **IHC** |  |  |  |  |  |
| HER2 Mem H‑score |  |  |  |  |  |
| ER-negative | High | 14/34 (41.18%) | 28/40 (70.00%) | 8/33 (24.24%) | 9/31 (29.03%) |
|  | Low | 9/24 (37.50%) | 10/21 (47.62%) | 7/24 (29.17%) | 7/22 (31.82%) |
|  | Chi-square (FDR) | 0.74 (1.00) | 0.09 (0.91) | 0.86 (1.00) | 0.77 (1.00) |
| ER-positive | High | 3/20 (15.00%) | 8/17 (47.06%) | 2/19 (10.53%) | 4/20 (20.00%) |
|  | Low | 5/28 (17.86%) | 3/29 (10.34%) | 1/30 (3.33%) | 3/23 (13.04%) |
|  | Chi-square (FDR) | 0.81 (1.00) | 0.01 (0.64) | 0.33 (0.94) | 0.59 (1.00) |
| HER3 Mem H‑score |  |  |  |  |  |
| ER-negative | High | 8/19 (42.11%) | 20/32 (62.50%) | 6/25 (24.00%) | 9/24 (37.50%) |
|  | Low | 14/36 (38.89%) | 16/26 (61.54%) | 9/30 (30.00%) | 6/21 (28.57%) |
|  | Chi-square (FDR) | 0.94 (1.00) | 0.94 (1.00) | 0.76 (1.00) | 0.65 (1.00) |
| ER-positive | High | 4/32 (12.50%) | 5/23 (21.74%) | 1/32 (3.13%) | 2/20 (10.00%) |
|  | Low | 3/12 (25.00%) | 5/15 (33.33%) | 1/12 (8.33%) | 3/18 (16.67%) |
|  | Chi-square (FDR) | 0.32 (0.94) | 0.71 (1.00) | 0.37 (0.94) | 0.58 (1.00) |
| pAKT Cyt  H-score |  |  |  |  |  |
| ER-negative | High | 10/23 (43.48%) | 24/34 (70.59%) | 6/30 (20.00%) | 9/24 (37.50%) |
|  | Low | 4/13 (30.77%) | 7/14 (50.00%) | 3/12 (25.00%) | 6/13 (46.15%) |
|  | Chi-square (FDR) | 0.48 (1.00) | 0.19 (0.94) | 0.87 (1.00) | 0.66 (1.00) |
| ER-positive | High | 4/24 (16.67%) | 4/17 (23.53%) | 0/23 (0.00%) | 5/22 (22.73%) |
|  | Low | 1/12 (8.33%) | 3/13 (23.08%) | 0/14 (0.00%) | 0/11 (0.00%) |
|  | Chi-square (FDR) | 0.42 (0.96) | 0.79 (1.00) | – | 0.10 (0.91) |
| IGF1R Mem H-score |  |  |  |  |  |
| ER-negative | High | 9/15 (60.00%) | 11/18 (61.11%) | 5/21 (23.81%) | 7/14 (50.00%) |
|  | Low | 12/36 (33.33%) | 20/29 (68.97%) | 5/26 (19.23%) | 8/26 (30.77%) |
|  | Chi-square (FDR) | 0.15 (0.91) | 0.59 (1.00) | 0.65 (1.00) | 0.23 (0.94) |
| ER-positive | High | 6/31 (19.35%) | 7/25 (28.00%) | 1/35 (2.86%) | 4/27 (14.81%) |
|  | Low | 1/6 (16.67%) | 1/10 (10.00%) | 0/8 (0.00%) | 3/11 (27.27%) |
|  | Chi-square (FDR) | 0.87 (1.00) | 0.21 (0.94) | 0.53 (1.00) | 0.35 (0.94) |
| PTEN Cyt  H-score |  |  |  |  |  |
| ER-negative | High | 13/34 (38.24%) | 29/42 (69.05%) | 10/33 (30.30%) | 9/26 (34.62%) |
|  | Low | 9/19 (47.37%) | 5/14 (35.71%) | 4/20 (20.00%) | 7/18 (38.89%) |
|  | Chi-square (FDR) | 0.45 (0.99) | 0.03 (0.82) | 0.36 (0.94) | 1.00 (1.00) |
| ER-positive | High | 7/36 (19.44%) | 8/36 (22.22%) | 1/31 (3.23%) | 5/31 (16.13%) |
|  | Low | 0/6 (0.00%) | 1/3 (33.33%) | 0/13 (0.00%) | 2/10 (20.00%) |
|  | Chi-square (FDR) | 0.22 (0.94) | 0.88 (1.00) | 0.48 (1.00) | 0.80 (1.00) |
| pAKT Nuc  H-score |  |  |  |  |  |
| ER-negative | High | 7/17 (41.18%) | 16/25 (64.00%) | 5/23 (21.74%) | 7/19 (36.84%) |
|  | Low | 7/19 (36.84%) | 15/23 (65.22%) | 4/19 (21.05%) | 8/18 (44.44%) |
|  | Chi-square (FDR) | 0.72 (1.00) | 0.94 (1.00) | 0.76 (1.00) | 0.70 (1.00) |
| ER-positive | High | 1/14 (7.14%) | 5/16 (31.25%) | 0/18 (0.00%) | 5/20 (25.00%) |
|  | Low | 4/22 (18.18%) | 2/14 (14.29%) | 0/19 (0.00%) | 0/13 (0.00%) |
|  | Chi-square (FDR) | 0.41 (0.96) | 0.23 (0.94) | – | 0.07 (0.88) |
| PTEN Nuc  H-score |  |  |  |  |  |
| ER-negative | High | 12/33 (36.36%) | 15/26 (57.69%) | 8/22 (36.36%) | 9/18 (50.00%) |
|  | Low | 10/20 (50.00%) | 19/30 (63.33%) | 6/31 (19.35%) | 7/26 (26.92%) |
|  | Chi-square (FDR) | 0.35 (0.94) | 0.65 (1.00) | 0.13 (0.91) | 0.12 (0.91) |
| ER-positive | High | 3/20 (15.00%) | 7/23 (30.43%) | 0/21 (0.00%) | 3/24 (12.50%) |
|  | Low | 4/22 (18.18%) | 2/16 (12.50%) | 1/23 (4.35%) | 4/17 (23.53%) |
|  | Chi-square (FDR) | 0.77 (1.00) | 0.14 (0.91) | 0.29 (0.94) | 0.36 (0.94) |
| **Serum** |  |  |  |  |  |
| Amphiregulin (pg/mL) |  |  |  |  |  |
| ER-negative | High | 10/25 (40.00%) | 14/26 (53.85%) | 7/25 (28.00%) | 6/20 (30.00%) |
|  | Low | 11/29 (37.93%) | 21/29 (72.41%) | 8/30 (26.67%) | 9/26 (34.62%) |
|  | Chi-square (FDR) | 0.94 (1.00) | 0.16 (0.92) | 0.87 (1.00) | 0.89 (1.00) |
| ER-positive | High | 7/28 (25.00%) | 7/21 (33.33%) | 2/24 (8.33%) | 5/24 (20.83%) |
|  | Low | 0/15 (0.00%) | 4/23 (17.39%) | 1/21 (4.76%) | 2/17 (11.76%) |
|  | Chi-square (FDR) | 0.04 (0.82) | 0.35 (0.94) | 0.72 (1.00) | 0.38 (0.94) |
| EGF (pg/mL) |  |  |  |  |  |
| ER-negative | High | 12/24 (50.00%) | 18/30 (60.00%) | 7/28 (25.00%) | 6/19 (31.58%) |
|  | Low | 9/30 (30.00%) | 17/25 (68.00) | 8/27 (29.63%) | 9/27 (33.33%) |
|  | Chi-square (FDR) | 0.15 (0.91) | 0.54 (1.00) | 0.78 (1.00) | 0.95 (1.00) |
| ER-positive | High | 4/23 (17.39%) | 8/24 (33.33%) | 0/20 (0.00%) | 3/24 (12.5%) |
|  | Low | 3/20 (15.00%) | 3/20 (15.00%) | 3/25 (12.00%) | 4/17 (25.53%) |
|  | Chi-square (FDR) | 0.84 (1.00) | 0.13 (0.91) | 0.13 (0.91) | 0.38 (0.94) |
| TGFα (pg/mL) |  |  |  |  |  |
| ER-negative | High | 13/24 (54.17%) | 16/25 (64.00%) | 7/31 (22.58%) | 7/22 (31.82%) |
|  | Low | 8/30 (26.67%) | 19/30 (63.33%) | 8/24 (33.33%) | 8/24 (33.33%) |
|  | Chi-square (FDR) | 0.05 (0.82) | 0.96 (1.00) | 0.42 (0.96) | 0.99 (1.00) |
| ER-positive | High | 5/25 (20.00%) | 7/20 (35.00%) | 0/25 (0.00%) | 3/20 (15.00%) |
|  | Low | 2/18 (11.11%) | 4/24 (16.67%) | 3/20 (15.00%) | 4/21 (19.05%) |
|  | Chi-square (FDR) | 0.44 (0.99) | 0.24 (0.94) | 0.01 (0.64) | 0.74 (1.00) |
| sHER2 (ng/mL) |  |  |  |  |  |
| ER-negative | High | 10/29 (34.48%) | 18/31 (58.06%) | 11/31 (35.48%) | 10/31 (32.26%) |
|  | Low | 11/25 (44.00%) | 17/24 (70.83%) | 4/24 (16.67%) | 5/15 (33.33%) |
|  | Chi-square (FDR) | 0.28 (0.94) | 0.34 (0.94) | 0.09 (0.91) | 0.98 (1.00) |
| ER-positive | High | 4/17 (23.53%) | 3/15 (20.00%) | 1/15 (6.67%) | 3/22 (13.64%) |
|  | Low | 3/26 (11.54%) | 7/27 (25.93%) | 2/29 (6.90%) | 4/19 (21.05%) |
|  | Chi-square (FDR) | 0.27 (0.94) | 0.72 (1.00) | 0.89 (1.00) | 0.60 (1.00) |
| **qRT-PCR** |  |  |  |  |  |
| *Amphiregulin* CR |  |  |  |  |  |
| ER-negative | High | 9/17 (52.94%) | 12/23 (52.17%) | 7/20 (35.00%) | 6/17 (35.29%) |
|  | Low | 9/30 (30.00%) | 19/29 (65.52%) | 7/28 (25.00%) | 9/25 (36.00%) |
|  | Chi-square (FDR) | 0.14 (0.91) | 0.33 (0.94) | 0.49 (1.00) | 0.83 (1.00) |
| ER-positive | High | 8/27 (29.63%) | 7/27 (25.93%) | 1/26 (3.85%) | 4/22 (18.18%) |
|  | Low | 0/18 (0.00%) | 2/11 (18.18%) | 1/20 (5.00%) | 3/18 (16.67%) |
|  | Chi-square (FDR) | 0.01 (0.64) | 0.79 (1.00) | 0.92 (1.00) | 0.89 (1.00) |
| *Betacellulin* CR |  |  |  |  |  |
| ER-negative | High | 7/18 (38.89%) | 15/25 (60.00%) | 7/22 (31.82%) | 6/19 (31.58%) |
|  | Low | 12/28 (42.86%) | 14/24 (58.33%) | 5/21 (23.81%) | 8/20 (40.00%) |
|  | Chi-square (FDR) | 0.74 (1.00) | 0.90 (1.00) | 0.56 (1.00) | 0.60 (1.00) |
| ER-positive | High | 2/20 (10.00%) | 3/16 (18.75%) | 1/23 (4.35%) | 4/19 (21.05%) |
|  | Low | 3/18 (16.67%) | 6/17 (35.29%) | 1/18 (5.56%) | 2/16 (12.50%) |
|  | Chi-square (FDR) | 0.56 (1.00) | 0.17 (0.92) | 0.76 (1.00) | 0.51 (1.00) |
| *EGFR* CR |  |  |  |  |  |
| ER-negative | High | 11/25 (44.00%) | 21/37 (56.76%) | 10/32 (31.25%) | 7/21 (33.33%) |
|  | Low | 10/28 (35.71%) | 13/19 (68.42%) | 4/20 (20.00%) | 9/25 (36.00%) |
|  | Chi-square (FDR) | 0.49 (1.00) | 0.41 (0.96) | 0.38 (0.94) | 0.99 (1.00) |
| ER-positive | High | 2/17 (11.76%) | 3/14 (21.43%) | 1/22 (4.55%) | 4/21 (19.05%) |
|  | Low | 6/26 (23.08%) | 7/27 (25.93%) | 1/25 (4.00%) | 3/18 (16.67%) |
|  | Chi-square (FDR) | 0.35 (0.94) | 0.83 (1.00) | 0.85 (1.00) | 0.94 (1.00) |
| *HER2* CR |  |  |  |  |  |
| ER-negative | High | 15/31 (48.39%) | 22/33 (66.67%) | 11/30 (36.67%) | 11/26 (42.31%) |
|  | Low | 6/23 (26.09%) | 13/24 (54.17%) | 3/24 (12.50%) | 5/20 (25.00%) |
|  | Chi-square (FDR) | 0.11 (0.91) | 0.35 (0.94) | 0.05 (0.82) | 0.23 (0.94) |
| ER-positive | High | 5/22 (22.73%) | 6/17 (35.29%) | 0/17 (0.00%) | 3/19 (15.79%) |
|  | Low | 3/24 (12.50%) | 4/25 (16.00%) | 3/31 (9.68%) | 4/21 (19.05%) |
|  | Chi-square (FDR) | 0.32 (0.94) | 0.22 (0.94) | 0.21 (0.94) | 0.83 (1.00) |
| *HER2/HER3* CR |  |  |  |  |  |
| ER-negative | High | 16/39 (41.03%) | 23/38 (60.53%) | 11/30 (36.67%) | 12/29 (41.38%) |
|  | Low | 5/14 (35.71%) | 12/19 (63.16%) | 3/24 (12.50%) | 4/17 (23.53%) |
|  | Chi-square (FDR) | 0.58 (1.00) | 0.85 (1.00) | 0.03 (0.82) | 0.26 (0.94) |
| ER-positive | High | 2/16 (12.50%) | 6/17 (35.29%) | 1/11 (9.09%) | 3/12 (25.00%) |
|  | Low | 6/29 (20.69%) | 4/24 (16.67%) | 2/37 (5.41%) | 4/28 (14.29%) |
|  | Chi-square (FDR) | 0.51 (1.00) | 0.26 (0.94) | 1.00 (1.00) | 0.45 (0.99) |
| *HER3* CR |  |  |  |  |  |
| ER-negative | High | 7/16 (43.75%) | 14/23 (60.87%) | 7/23 (30.43%) | 7/19 (36.84%) |
|  | Low | 14/37 (37.84%) | 21/34 (61.76%) | 7/31 (22.58%) | 9/27 (33.33%) |
|  | Chi-square (FDR) | 0.84 (1.00) | 0.95 (1.00) | 0.57 (1.00) | 0.78 (1.00) |
| ER-positive | High | 5/29 (17.24%) | 5/24 (20.83%) | 0/30 (0.00%) | 5/28 (17.86%) |
|  | Low | 3/16 (18.75%) | 5/17 (29.41%) | 3/18 (16.67%) | 2/12 (16.67%) |
|  | Chi-square (FDR) | 0.98 (1.00) | 0.57 (1.00) | 0.06 (0.82) | 0.89 (1.00) |
| **FISH** |  |  |  |  |  |
| Target:centromere ratio (*c-Myc*) |  |  |  |  |  |
| ER-negative | High | 3/10 (30.00%) | 8/12 (66.67%) | 2/12 (16.67%) | 1/10 (10.00%) |
|  | Low | 16/32 (50.00%) | 15/27 (55.56%) | 4/20 (20.00%) | 10/23 (43.48%) |
|  | Chi-square (FDR) | 0.18 (0.94) | 0.53 (1.00) | 0.86 (1.00) | 0.06 (0.82) |
| ER-positive | High | 3/9 (33.33%) | 4/12 (33.33%) | 0/11 (0.00%) | 2/11 (18.18%) |
|  | Low | 3/23 (13.04%) | 4/17 (23.53%) | 2/24 (8.33%) | 3/22 (13.64%) |
|  | Chi-square (FDR) | 0.17 (0.92) | 0.60 (1.00) | 0.30 (0.94) | 0.78 (1.00) |
| **SNP** |  |  |  |  |  |
| *PIK3CA*  (*any mutation*) |  |  |  |  |  |
| ER-negative | WT | 13/23 (56.52%) | 19/32 (59.38%) | 6/25 (24.00%) | 10/25 (40.00%) |
|  | Mut | 4/15 (26.67%) | 6/10 (60.00%) | 2/8 (25.00%) | 2/8 (25.00%) |
|  | Chi-square (FDR) | 0.12 | 0.95 | 0.95 | 0.40 |
| ER-positive | WT | 2/20 (10.00%) | 6/17 (35.29%) | 2/25 (8.00%) | 4/18 (22.22%) |
|  | Mut | 2/12 (16.67%) | 1/10 (10.00%) | 0/13 (0.00%) | 2/12 (16.67%) |
|  | Chi-square | 0.63 | 0.27 | 0.33 | 0.93 |
|  |  |  |  |  |  |

CR, concentration ratio; Cyt, cytoplasmic; EGF, epidermal growth factor; EGFR, epidermal growth factor receptor; ELISA, enzyme-linked immunosorbent assay; FISH, fluorescence *in situ* hybridization; IGF1R, insulin-like growth factor 1 receptor; IHC, immunohistochemistry; Mem, membranous; Mut, mutant; Nuc, nuclear; pCR, pathologic complete response; *PIK3CA*, gene encoding phosphoinositide 3-kinase, catalytic subunit; PTEN, phosphatase and tensin homolog; qRT-PCR, quantitative reverse transcription polymerase chain reaction; sHER2, serum HER2 extracellular domain; TGF, transforming growth factor; WT, wild-type.

Cochran–Mantel–Haenszel Chi-square test based on biomarker subgroup x pCR status (2x2) stratified by breast cancer type.
