# Supplementary material for: Biomarker analysis of the NeoSphere study: pertuzumab, trastuzumab, and docetaxel versus trastuzumab plus docetaxel, pertuzumab plus trastuzumab, or pertuzumab plus docetaxel for the neoadjuvant treatment of HER2-positive breast cancer
Source: Breast Cancer Res. 2017 Feb 9;19:16. doi: 10.1186/s13058-017-0806-9 (PMC5299741; doi:10.1186/s13058-017-0806-9)
Supplement: Additional file 2: — Figure S1. Relationship between biomarkers and pCR by treatment group and estrogen receptor status. CR concentration ratio, Cyt cytoplasmic, EGF epidermal growth factor, EGFR epidermal growth factor receptor, ELISA enzyme-linked immunosorbent assay, FISH fluorescence in situ hybridization, IGF1R insulin-like growth factor 1 receptor, IHC immunohistochemistry, Mem membranous, Nuc nuclear, pCR pathologic complete response, PIK3CA gene encoding phosphoinositide 3-kinase, catalytic subunit; PTEN phosphatase and tensin homolog, qRT-PCR quantitative reverse transcription PCR, sHER2 serum HER2 extracellular domain, TGF transforming growth factor; WT wild-type. (PDF 836 kb) [file 13058_2017_806_MOESM2_ESM.pdf]

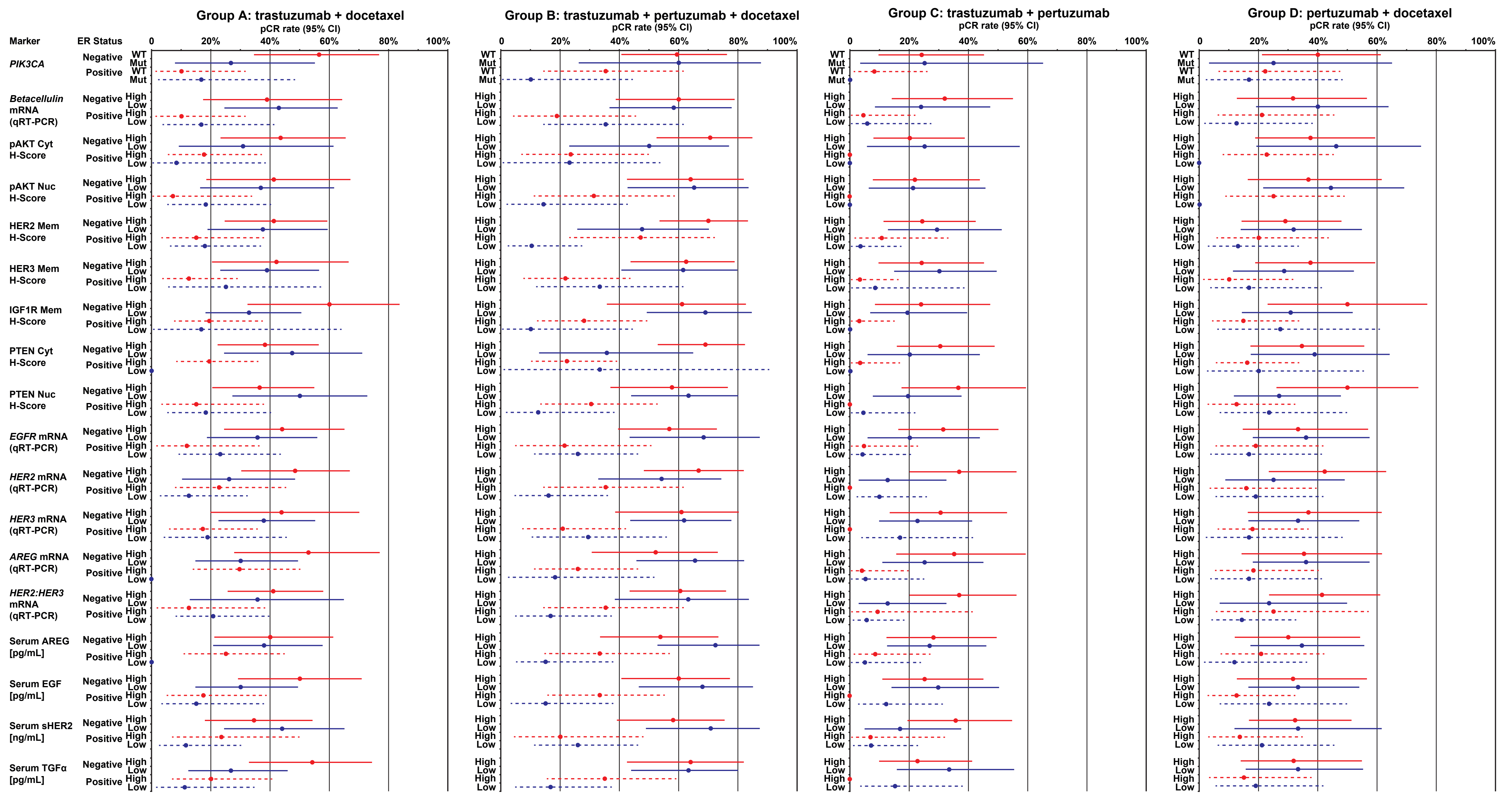

Group A: trastuzumab + docetaxel
Group B: trastuzumab + pertuzumab + docetaxel
Group C: trastuzumab + pertuzumab
Group D: pertuzumab + docetaxel
pCR rate (95% CI)
Marker
ER Status
0
20%
40%
60%
80%
100%
PIK3CA
Betacellulin mRNA (qRT-PCR)
pAKT Cyt H-Score
pAKT Nuc H-Score
HER2 Mem H-Score
HER3 Mem H-Score
IGF1R Mem H-Score
PTEN Cyt H-Score
PTEN Nuc H-Score
EGFR mRNA (qRT-PCR)
HER2 mRNA (qRT-PCR)
HER3 mRNA (qRT-PCR)
AREG mRNA (qRT-PCR)
HER2:HER3 mRNA (qRT-PCR)
Serum AREG [pg/mL]
Serum EGF [pg/mL]
Serum sHER2 [ng/mL]
Serum TGFα [pg/mL]
Negative
Positive
WT
Mut
High
Low
